# Substrate Factors Determine Roadside Vegetation Structure and Species Richness: A Case Study Along a Meridional Gradient in Fennoscandia

**DOI:** 10.1007/s00128-016-1895-3

**Published:** 2016-08-02

**Authors:** Małgorzata Jaźwa, Waldemar Heise, Beata Klimek

**Affiliations:** 1 Institute of Botany, Faculty of Biology and Earth Sciences, Jagiellonian University, Kopernika 27, 31-501 Kraków, Poland; 2 Institute of Environmental Sciences, Faculty of Biology and Earth Sciences, Jagiellonian University, Gronostajowa 7, 30-387 Kraków, Poland

**Keywords:** Ecological corridors, Road ecology, Salt deposition, Substrate, Traffic system

## Abstract

This study assessed the effects of road-related alteration of substrate, including increased salinity, on vegetation along a meridional gradient in Fennoscandia. Vegetation community composition were surveyed in 29 randomly selected 1-m^2^ sized roadside plots. Number of plant species and plant cover (%) on the plots were positively interrelated (*p* < 0.0001). Both variables also decreased towards the north and with increasing coarseness of the substrate. Canonical correspondence analysis (CCA) indicated that roadside vegetation diversity and composition were most related to the importance of the road (i.e. its size and traffic intensity) and substrate pH. Road importance affects plant dispersal, whereas substrate pH was found to be a factor limiting growth. CCA indicated also that vegetation composition was affected by the meridional gradient and by the substrate salinity; both substrate salinity pH and salinity were not related to meridional gradient. Our results indicate that roadside vegetation diversity and composition is driven by natural and anthropogenic factors.

Alteration of soil properties along roads is assumed to have a negative impact on vegetation diversity and structure (Brown and Gorres 2011; Müllerová et al. 2011; Malinowska et al. 2015). The synergistically operating factors influencing plant growth include high insolation and extreme temperatures along tarmac roads, high wind speed, low organic matter content, increased levels of road-use-related contamination (e.g. salinity) and continuous anthropopression (mowing and cutting of vegetation) (Müllerová et al. 2011; Fan et al. 2014). The high level of environmental stress typical for roadsides leads to the development of specific plant communities.

Roads, being a major human promoter of urbanisation, allow for plants to be dispersed by transportation and road building materials. Roads are ecological corridors which support the expansion of a taxon’s geographical distribution (Hayasaka et al. 2012). There is growing interest in the effects of roads on the dispersal and expansion of plants, especially non-native ones (Hayasaka et al. 2012; Tyler et al. 2015). The sources of alien plants include motor vehicle traffic and the movement of seed banks in road-building material or in the gravel applied in winter to minimize skidding. Alien plants may enrich the local flora but may also cause an invasion, potentially altering ecosystem processes and functions (Hayasaka et al. 2012; Watkins et al. 2003). Another source of alien plants, or of plants not typical for the surrounding habitat, is routine sowing of fast-growing grassland species on road verges (Tikka et al. 2000).

The increased climate harshness along a meridional gradient is one of the most important factors determining vegetation diversity and composition (Mannion et al. 2014). Temperature, insolation, precipitation, and the seasonal distribution of these factors determine decreased alpha plant diversity (number of species) and change beta plant diversity (species composition) towards north (Mannion et al. 2014).

Among the different physical and chemical properties of the substrate, pH and salinity seem to be the most important factors shaping the composition of roadside vegetation. Alkaline gravel, often used on roads, may change substrate pH in the near vicinity, especially in nutrient-poor environments (Müllerová et al. 2011). This can alter the plant species composition, favouring species that prefer higher substrate pH (Rose and Hermanutz 2004). Roadside vegetation is often exposed to higher salinity due to the use of de-icing agents, mainly sodium chloride (NaCl) and calcium magnesium acetate (CaMg_2_(CH_3_COO)_6_). For road managements with seasonal climate, road salt is used along fine gravel to improve road safety in hazardous icy conditions in winter. Salinity is one of the strongest environmental factors limiting plant growth and productivity (Allakhverdiev et al. 2000). Plants that can survive and grow well under high salinity in the rhizosphere are called halophytes; such plants employ many defence mechanisms (Parida and Dasa 2005; Skorupa et al. 2016). Many plant species can tolerate increased salinity, but at the expense of their vigour. Fan et al. (2014) showed that de-icing road salts applied in the Sierra Nevada Mountains caused increased forest mortality in the vicinity (<10 m) of roads.

Our study examined vegetation on roadsides in the cold climate of Fennoscandia. Most of traffic-related emissions have clearly fallen there since the early 1980s (see data published by European Environment Agency, e.g. SOER 2015). Thus, we anticipated low levels of traffic associated contamination, which were, however, not further quantified. Although the human impact on the environment in Northern Europe is low relative to densely populated regions, the expected future climatic warming and changes in land use may soon lead to a rapid transformation of boreal and sub-arctic ecosystems, with species-poor boreal ecosystems being particularly vulnerable to various disturbances (Rose and Hermanutz 2004; Bradshaw et al. 2009; Węgrzyn et al. 2013). Knowledge of the qualitative characteristics of roadside vegetation can help clarify the mechanisms of floristic homogenisation resulting from long-distance transport of propagules over land. Such data can also serve as a baseline for tracking temporal changes in vegetation.

Roadsides may foster the formation of similar vegetation communities at sites remote from each other. In this study we surveyed roadside vegetation in the northern part of the Scandinavian Peninsula, and related the physicochemical characteristics of roadside substrate to the vegetation composition along a north–south gradient.

## Materials and Methods

**Fig. 1:**
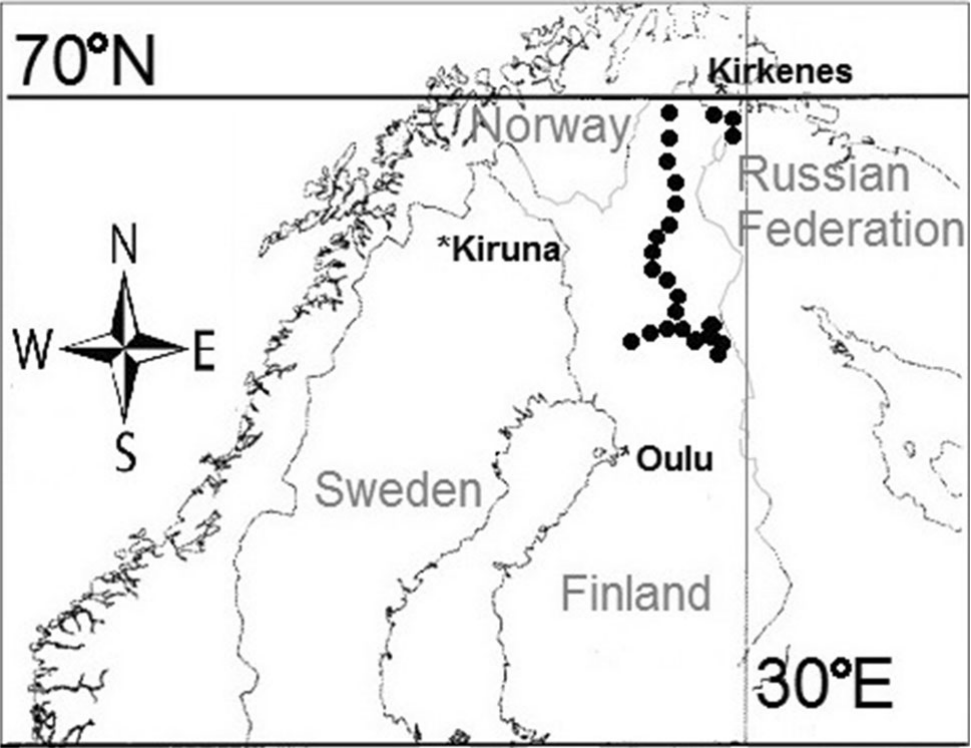
Distribution of studied plots (*filled dots*) in northern Fennoscandia

Study plots were randomly selected along several roads leading throughout part of the Scandinavian Peninsula (Finland and Norway). Figure 1 shows the distribution of the 29 study plots. The roads were classified in three groups according to size and traffic load: (1) highways and national roads, (2) local roads (tarmac), and (3) other local roads, including paved roads. In each study plot we designated representative roadside vegetation patches (1 m^2^) and made relevés there. Species were identified in the field, and the coverage of particular species on the study plot was recorded as percentage of plot area, meaning 100 % as area total coverage and 0 % as lack of coverage. Some plant species were identified only to genus level (*Peltigera* sp., *Salix* sp.). Means and standard deviations were calculated for number of plant species and plant cover for all studied plots.

Material was collected from roadsides and classified as road building material (aggregate, gravel, chippings) enriched with organic matter, mainly plant detritus. A sample was taken from the upper substrate layer (to 10 cm depth) at the centre of the relevé in each study plot. To compare substrate characteristics between the roadside and its vicinity, soil was also collected at three other distances from the road (5, 10, 150 m). Substrate and soil were collected to separate plastic bags and transported to the laboratory. The collected material was sieved (1 cm mesh) and stored field-moist at 4°C before further analyses.

The density of substrate and soil was determined as the weight of known volume of dry material. Dry weight (DW) was determined after drying of the substrate samples at 105°C for 24 h, and organic matter (OM) content was determined as the loss on ignition at 550°C for 24 h. Water holding capacity (WHC) was measured by a gravimetric method after soaking the substrate and soil for 24 h in net-ended plastic pipes immersed in water. Substrate and soil pH was measured in air-dried subsamples (3 g) shaken in water (1:10 w/v) for 1 h at 200 rpm. Substrate and soil conductivity (µmhos cm^−1^) was determined in the water slurry and converted to conductivity value at 25°C and then expressed as actual salinity per organic matter unit.

Fine-fraction subsamples of the roadside substrate were separated for elements concentration analysis and the final result was recalculated per total sample weight. The total C and N contents were analysed in fine-ground dry material with a CHNS analyser (Elementar Analysensysteme GmbH). The C:N, C:P and C:S ratios were calculated for each sample. Total element concentrations in each sample (Ca, K, Mg, Mn, Na, and P) were determined after wet digestion of 0.5 g DW of substrate in 10 mL of SupraPure HNO_3_ and HClO_4_ (7:1 v/v) (Sigma-Aldrich). Elements concentrations were measured by atomic absorption spectrometry (AAS, Perkin Elmer) with a flame (type AAnalyst200) or graphite furnace (type AAnalyst200) nebuliser, depending on concentration). Only the P concentration was measured with a flow-injection analyser (MLE Gmbh Dresnen, type FIA compact). To control accuracy, four blank samples and three replicates of standard certified material (CRM025-050, Sandy Loam 8, RT Corp.) were analysed with the substrate and soil samples.

One-way ANOVA was performed to test the significance of differences in means for substrate and soil physicochemical characteristics between the studied road distances. Normality of the data distribution within groups was assessed using the Shapiro–Wilk test and the data were transformed if necessary. Differences were considered statistically significant at *p* < 0.05. Pairwise differences in means were tested with Tukey’s test. Simple regressions were performed to assess the relation between substrate density (g cm^−3^ DW) and salinity (expressed as mg g^−1^ OM) as a proxy of salinity stress to plant roots. We checked also the relationships between these substrate properties and latitude.

Simple regression was performed to assess the relationship between number of plant species and plant cover (*p* < 0.05). Canonical correspondence analysis (CCA) was used to examine the correlation between vegetation composition in the 29 studied plots with latitude, road traffic character, and physicochemical properties of substrate. The final set of factors giving the best fit of the CCA model to vegetation data was chosen and checked for relationship with latitude with simple regressions. Additionally, simple regressions were performed to relate the number of plant species and plant cover (%) to the variables used in CCA analysis and to relate substrate pH and salinity with their location along meridional gradient.

The ANOVA and regression analyses were done with Statgraphics Centhurion XVI (StatPoint Technologies Inc., Warrenton VA, U.S.A.), and the CCA analyses used PAST 2.17c (Natural History Museum, University of Oslo, Norway).

## Results and Discussion

We identified 64 plant species on the 29 studied plots. The number of species per plot ranged from 2 to 15 (mean 8.1, S.D. 3.4). Scots pine (*Pinus sylvestris* L.) was the most common species, found on 19 of the plots. Only small pine seedlings were detected on roadsides (<15 cm of main shoot length). Twenty-two of the plant species were found only on single plots (e.g. *Equisetum arvense*, *Plantago maritima*, *Orthilia secunda*). The roadside vegetation was composed mainly of species with a wide ecological amplitude, such as the commonly sown grass *Festuca ovina* or dwarf forms of *Pinus sylvestris*. The presence of species typical for wetlands (e.g. *Parnassia palustris*, *Ledum palustre*) reflected the fact that some roads were constructed near bogs or even across them (as levees), or along roadside ditches. The majority of species occurring in single stands were common boreal zone species, but the presence of a *Plantago maritima* stand is especially interesting. *Plantago maritima* is typical for seaside, but this species was found at a stand 180 km from the coast, suggesting a large role of roads in the dispersal of this plant.

It is thought that boreal ecosystems are susceptible to alien plants invasions, especially in anthropogenically disturbed habitats (Rose and Hermanutz 2004). Increased soil pH is considered a factor fostering such invasions (Rose and Hermanutz 2004), especially in boreal acid podsolic soils with low calcium content (Ranta et al. 2015). However, given the type of area we studied, the absence of invasive alien plant species in the 29 randomly selected plots may suggest that northern Fennoscandia has a low rate of alien species invasion, yet.

**Fig. 2:**
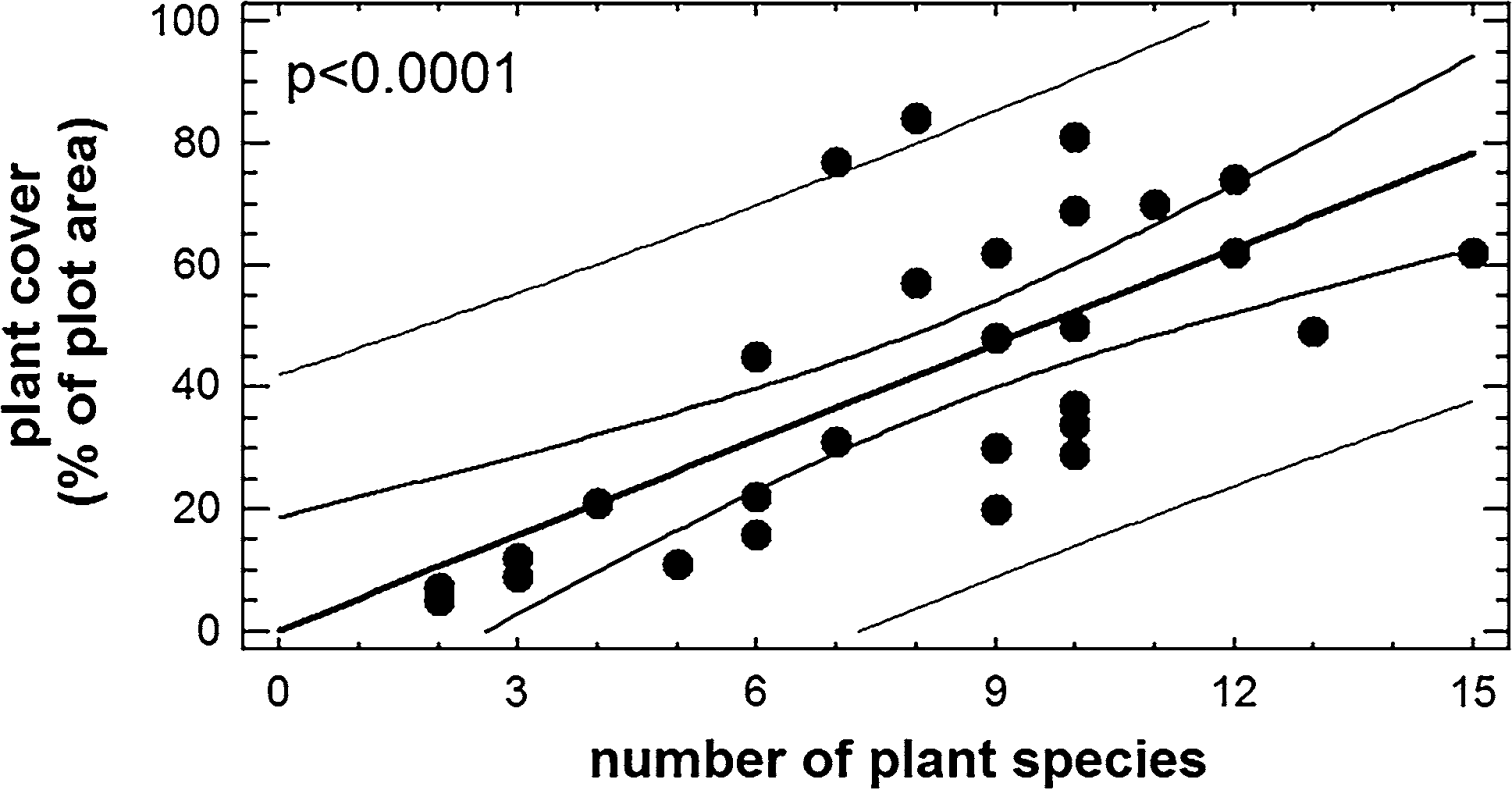
Relationship between number of plant species per plot and plant cover (% of plot area)

The mean plant cover in the relevés ranged from 5 % to 84 % (mean 43.0 %, S.D. 25.1 %). *Festuca ovina gg.*, *Empetrum nigrum* and *Betula pendula* had the highest cover on all plots if taken together. Also common were *Trifolium pratense* and *Trifolium repens*, species known to be resistant to physical damage, which are a frequent component of seed mixtures sown on road verges (Tikka et al. 2000). There was a highly significant positive relationship between number of plant species and plant cover (*p* < 0.0001; r = 0.70) (Fig. 2). Tilman et al. (1997) observed a similar relationship in their classical study of grasslands; however, they related plant diversity to the plant biomass, which is not directly linked to plant cover.

Substrate density was highest in samples from roadsides, indicating a higher fraction of inorganic matter than in samples collected further from the road (Table 1). The organic matter content of roadside substrate was very low, as was its water holding capacity (Table 1). Substrate pH and salinity were higher on roadsides that at sites further from the road (Table 1). Higher substrate pH on roadsides, also found by other authors, may result from the use of alkaline gravel in road construction (Černohlávková et al. 2008). Simple regressions indicated a strong positive correlation between substrate density (g cm^−3^) and salinity (g kg^−1^ OM) (*p* < 0.0001; r = 0.64), confirming that salinity is linked to the composition of road-building material and road maintenance practices. Both substrate pH and salinity on roadsides were not related to meridional gradient (*p* = 0.5268 and *p* = 0.7895, respectively). Salinity is a strong stressor for roadside vegetation as well as for soil organisms, including microorganisms beneficial to plants (Černohlávková et al. 2008). However, substrate density was related to meridional gradient and the higher latitude, the higher substrate density (coarser substrate structure) was found (*p* = 0.0295). The coarser substrate under northward stands is attributable to lower organic matter content (less plant detritus) on roadsides. Lower plant growth and plant size may be due to the colder climate accompanying the difference in habitat.

**Table 1:** Substrate and soil physicochemical characteristics of samples (OM denote organic matter content, WHC denote water holding capacity) collected at different distances from the road (mean ± S.D., n = 29)

Distance from the road (m)	Substrate and soil physicochemical characteristics					
	Density* (g cm^−3^)	OM* (% DW)	WHC* (%)	pH* (pH unit)	Salinity* (g kg^−1^ OM)	N-NO_3_^−^* (mg N kg^−1^ DW)
0	0.72^c^ (±0.25)	3.8^a^ (±3.8)	58.0^a^ (±42.3)	5.35^c^ (±0.30)	7.18^b^ (±5.58)	26.2^a^ (±50.5)
5	0.20^b^ (±0.17)	48.2^b^ (±25.6)	468.1^b^ (±296.1)	4.42^b^ (±0.47)	1.72^a^ (±1.16)	59.8^b^ (±78.7)
10	0.11^a^ (±0.06)	80.2^c^ (±16.4)	787.0^c^ (±418.7)	4.01^a^ (±0.40)	0.98^a^ (±0.71)	72.6^b^ (±73.1)
150	0.09^a^ (±0.04)	80.3^c^ (±17.5)	738.9^c^ (±283.0)	3.98^a^ (±0.43)	1.12^a^ (±0.79)	70.1^b^ (±59.1)

Asterisked variables differ significantly among distances (*p* < 0.05). Different small letters in superscripts indicate statistically significant differences between spots assessed at different distances from the road

Elements content and their ratios were lowest in roadside substrate (Table 2), showing that roadsides furnish an extremely poor environment for vegetation. The coarse structure of roadside substrate exposes it to washout and consequent depletion of biogens such as nitrogen and sulphur.

**Table 2:** Elements content and ratios in substrate and soil collected at different distances from the road (mean ±S.D., n = 29)

Distance from the road (m)	Elements content and their ratios					
	N* (% DW)	P* (% DW)	S* (% DW)	C:N*	C:P*	C:S*
0	0.06^a^ (±0.12)	0.02^a^ (±0.01)	0.02^a^ (±0.04)	29.2^a^ (±8.5)	78.6^a^ (±101.1)	98.6^a^ (±100.8)
5	0.87^b^ (±0.46)	0.06^b^ (±0.03)	0.07^b^ (±0.05)	29.3^a^ (±7.5)	396.6^b^ (±158.6)	387.9^b^ (±170.2)
10	1.26^c^ (±0.44)	0.08^bc^ (±0.03)	0.08^b^ (±0.05)	35.6^b^ (±7.5)	580.6^c^ (±145.8)	577.6^c^ (±283.4)
150	1.40^c^ (±0.55)	0.09^c^ (±0.04)	0.10^b^ (±0.04)	34.6^ab^ (±9.7)	561.2^c^ (±232.3)	533.9^c^ (±226.6)

Asterisked variables differ significantly between distances (*p* < 0.05). Different small letters in superscripts indicate statistically significant differences between spots assessed at different distances from the road

In turn, the concentrations of other essential elements (K, Na, Ca, Mg) were significantly higher in roadside substrate than in samples collected further from the road (Table 3). This result reflects the higher mineral fraction on roadsides than in samples collected further from the road (Černohlávková et al. 2008). The ‘road effect’ on substrate characteristics and element content disappeared between 5 and 10 m from the road; the majority of measured physicochemical characteristics did not differ between samples collected 10 m from the road and those collected 150 m from the road.

**Table 3:** Elements content in substrate and soil collected at different distances from the road (mean ±S.D., n = 29)

Distance from the road (m)	Elements content			
	K* (% DW)	Na* (% DW)	Mg** (% DW)	Ca* (% DW)
0	0.23^c^ (±0.11)	0.062^c^ (±0.02)	0.53^c^ (±0.25)	0.71^b^ (±0.30)
5	0.17^b^ (±0.06)	0.041^b^ (±0.03)	0.32^b^ (±0.17)	0.47^a^ (±0.25)
10	0.11^a^ (±0.04)	0.017^a^ (±0.01)	0.14^a^ (±0.11)	0.36^a^ (±0.17)
150	0.12^a^ (±0.04)	0.014^a^ (±0.01)	0.12^a^ (±0.08)	0.36^a^ (±0.16)

Asterisked values differ significantly between distances (*p* < 0.05). Different small letters in superscripts indicate statistically significant differences between spots assessed at different distances from the road

**Fig. 3:**
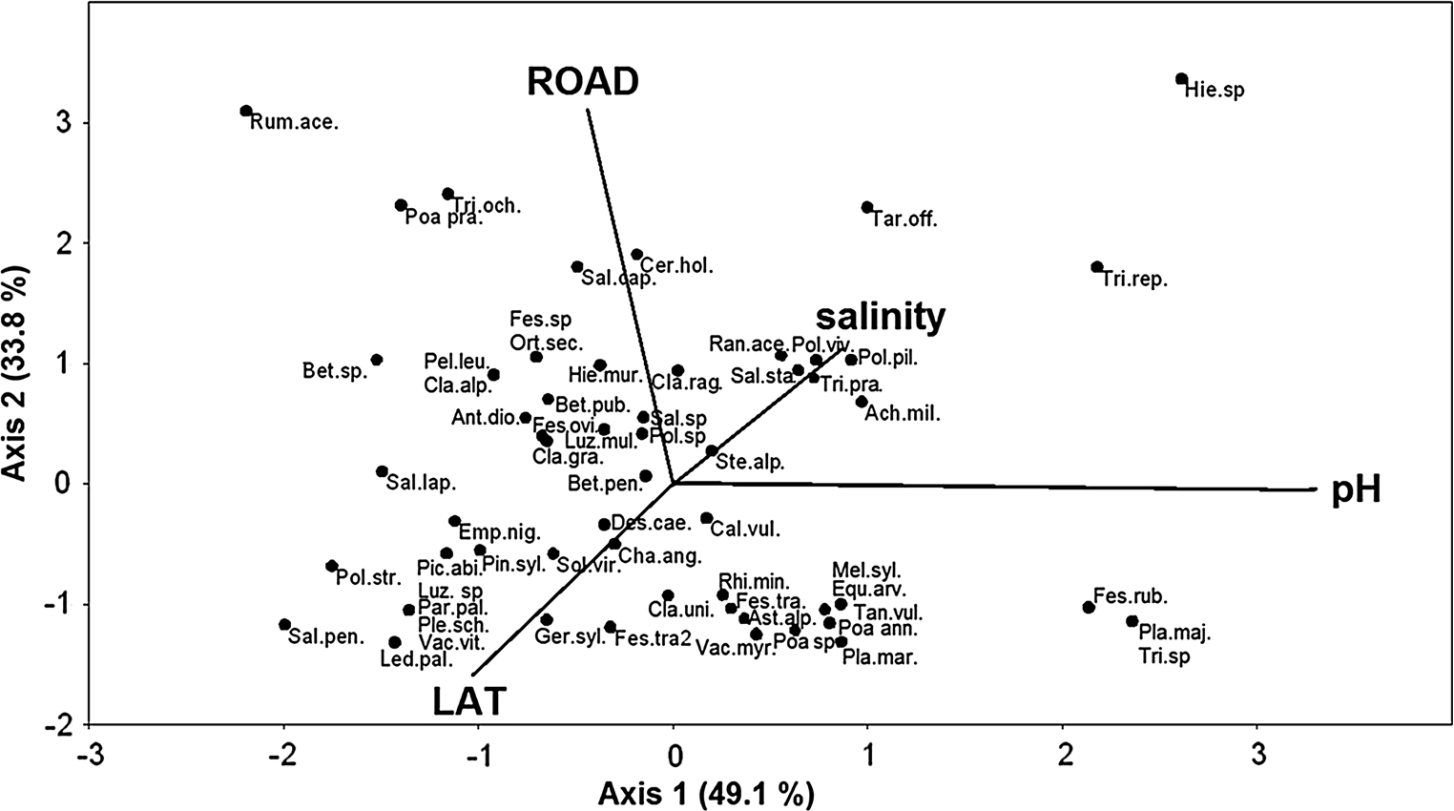
The canonical correspondence analysis (CCA) ordination plot of the vegetation diversity and composition according to site properties including latitude (LAT), road size and traffic intensity (ROAD), and substrate pH and salinity. Species are shown as dots with abbreviations. Abbreviation of species: Lichens and mosses: Cla.rag.—*Cladonia ragnifera*, Cla.uni.—*Cladonia uncialis*, Cla.alp.—*Cladonia alpestris*, Cla.gra.—*Cladonia gracilis*, Ple.sch.—*Pleurozium schreberi*, Pol.viv.—*Polygonum viviparum*, Pol.pil.—*Polytrichum piliferum*, Pol.sp—*Polytrichum* sp., Pol.str.—*Polytrichum strictum*, Ste.alp.—*Stereocaulon alpinum.* Vascular plants: Ach.mil.—*Achillea millefolium*, Ant.dio.—*Antennaria dioica*, Ast.alp.—*Astragalus alpinus*, Bet.pen.—*Betula pendula*, Bet.pub.—*Betula pubescens*, Bet.sp—*Betula* sp., Cal.vul.—*Calluna vulgaris*, Cer.hol.—*Cerastium holosteoides*, Cha.ang.—*Chamaenerion angustifolium*, Des.cae.—*Deschampsia caespitosa*, Des.fle. –*Deschampsia flexuosa*, Emp.nig.—*Empetrum nigrum*, Equ.arv.—*Equisetum arvense*, Fes.sp—*Festuca* sp., Fes.ovi.1.—*Festuca ovina s. s.*, Fes.ovi.2—*Festuca ovina agg.*, Fes.rub.—*Festuca rubra*, Fes.tra.—*Festuca trachyphylla*, Ger.syl.—*Geranium sylvaticum*, Hie.mur.—*Hieracium murorum*, Hie.sp—*Hieracium* sp., Led.pal.—*Ledum palustre*, Luz.mul.—*Luzula multiflora*, Luz.sp—*Luzula* sp., Mel.syl.—*Melampyrum sylvaticum*, Ort.sec.—*Orthilia secunda*, Par.pal.—*Parnassia palustris*, Pel.leu.—*Peltigera leucophlebia*, Pic.alb.—*Picea abies*, Pin.syl.—*Pinus sylvestris*, Pla.maj.—*Plantago major*, Pla.mar.—*Plantago maritima*, Poa ann.—*Poa annua*, Poapra.—*Poa pratensis*, Poasp—*Poa* sp., Ran.ace.—*Ranunculus acris*, Rhi.min.—*Rhinanthus minor*, Rum.ace.—*Rumex acetosella*, Sal.cap.—*Salix caprea*, Sal.lap.—*Salix lapponum*, Sal.pen.—*Salix pentandra*, Sal.sp—*Salix* sp., Sal.sta.—*Salix starkeana*, Sol.vig. –*Solidago virgaurea*, Tan.vul.—*Tanacetum vulgare*, Tar.off.—*Taraxacum officinalis*, Tri.och.—*Trifolium ochroleucum*, Tri.pra.—*Trifolium pratense*, Tri.rep.—*Trifolium repens*, Tri.sp—*Trifolium* sp., Vac.myr. –*Vaccinium myrtillus*, Vac.vit.—*Vaccinium vitis*–*idaea*

The final set of environmental variables data in CCA analysis included latitude, road traffic character, substrate pH and its salinity. None of physicochemical properties of substrate used in CCA was related to latitude as showed with simple regressions (*p* > 0.05). The first two CCA axes calculated for vegetation diversity and composition explained 41.9 % (*p* = 0.0495) and 33.8 % (*p* = 0.0198) of the variance respectively (trace *p* = 0.0099). The first CCA axis was strongly and positively related to pH (0.82) and slightly less to salinity (0.22), and negatively related to latitude (−0.26) and road category (−0.11) (Fig. 3). Road category had the highest relevancy on the second axis (0.77). The second CCA axis was also positively related to salinity (0.28), and negatively related to latitude (−0.40) and substrate pH (−0.01) (Fig. 3).

Road size and traffic intensity affected the roadside vegetation. Some plant species were found only along small local roads (*Rumex acetosella*, *Trifolium ochroleucum*, *Poa pratensis*; Fig. 3). This may be related to lower levels of physical damage to plants along small local roads, or to differences in road verge management there, as compared to highways. Such effect was reported by Forman and Alexander (1998) in their review on major ecological effects caused by roads.

Roadside vegetation diversity and composition were strongly related to substrate pH even though the variability of roadside substrate pH was low (Table 1). Such phenomenon was also found by Rola et al. (2015). In contrast, the effect of salinity on vegetation was relatively weak despite the high variability of salinity (Table 1; Fig. 3). Enhanced salinity level affects soil structure, fractions dispersion, permeability and osmotic potential, and leads to loss of soil stability and also to osmotic stress on vegetation. Salinity stress may affect plants through reduced soil microorganisms viability (Černohlávková et al. 2008). Moreover, high salinity from de-icing agents may mobilise heavy metals in the roadside environment, as shown in a Swedish study (Bäckström et al. 2004), and metals can be a significant factor limiting the emergence of plants cover along road (Bae et al. 2015). In our study, white clover *Trifolium repens* and red clover *Trifolium pratense* were found to be more tolerant than other plant species to increased salinity. These plants are generally thought to be resistant to environmental stress (Stoychev et al. 2013). *Achillea millefolium*, *Hieracium* sp., *Taraxacum officinalis* and some *Polytrichum* mosses were also found to be more resistant to substrate salinity. On the other hand, the location of these species on the CCA plot may also suggest a preference for warmer end of studied meridional gradient, as the effect of salinity on roadside vegetation diversity and structure was ran counter to latitude and substrate density (Fig. 3).

Roadside vegetation diversity and composition were related to latitude; there was north–south gradient of species occurrence (Fig. 3). Decreasing mean annual temperature between the outmost stands towards the north (from ca 2.5 to 0.5°C) may affect vegetation diversity and structure through exclusion of species with divergent or narrower ranges of ecological tolerance. This has been observed commonly for different groups of organisms in many ecosystems (Mannion et al. 2014).

Simple regressions relating number of plant species per plot and percentage of plot cover to the variables used in CCA analysis showed that only latitude affected those two parameters, and these relations were negative (r values −0.46 and −0.56, *p* values 0.0122 and 0.0015, respectively). In other words, regression analysis showed that plant species number decreased with the increase of climate harshness toward the north. Road traffic level and substrate pH and salinity, which both were not related to meridional gradient, had no direct effect on plant species number or plot cover, but these factors affected plant community composition (beta diversity). In turn, substrate density (coarser substrate structure) was positively related to latitude and these suggest that substrate density also affect negatively on plant species number and plot cover.

Summing up, here we showed that latitude (climate) and substrate density are factors determining plant alpha diversity on roadsides, whereas local conditions—traffic level, substrate pH and salinity—determine the community composition of roadside vegetation, that is, its beta diversity. Roadside vegetation in boreal regions is subjected to various disturbances, which affect them through various ways. We showed that substrate pH and traffic load are the most important factors shaping roadside vegetation in northern Fennoscandia.
